# Risk of Melanoma and Non-Melanoma Skin Cancer in Patients with Psoriasis and Psoriatic Arthritis Treated with Targeted Therapies: A Systematic Review and Meta-Analysis

**DOI:** 10.3390/ph17010014

**Published:** 2023-12-21

**Authors:** Marta Krzysztofik, Paweł Brzewski, Przemysław Cuber, Artur Kacprzyk, Aleksandra Kulbat, Karolina Richter, Tomasz Wojewoda, Wojciech M. Wysocki

**Affiliations:** 1 Department of Dermatology and Venereology, Stefan Zeromski Municipal Hospital, 31-913 Krakow, Poland; 2 Faculty of Medicine and Health Sciences, Andrzej Frycz Modrzewski Krakow University, 30-705 Krakow, Polandwwysocki@mp.pl (W.M.W.); 3 Department of Oncological Surgery, 5th Military Clinical Hospital in Kraków, 30-901 Krakow, Poland; 4 Doctoral School of Medical and Health Sciences, Jagiellonian University Medical College, 31-530 Krakow, Poland; 5 National Institute of Oncology, Maria Skłodowska-Curie Memorial, 02-781 Warsaw, Poland

**Keywords:** psoriasis, psoriatic arthritis, melanoma, skin cancer, biologics, targeted therapies

## Abstract

Targeted therapies represent major advancements in the treatment of chronic skin conditions such as psoriasis. While previous studies have shown an increased risk of melanoma and non-melanoma skin cancer (NMSC) in patients receiving TNF-α inhibitors, the risks associated with newer biologics (IL-12/23 inhibitors, IL-23 inhibitors, IL-17 inhibitors) and Janus kinase (JAK) inhibitors remain less known. Using a systematic and meta-analytical approach, we aimed to summarize the currently available literature concerning skin cancer risk in patients treated with targeted therapies. The MEDLINE/PubMed, EMBASE, Web of Science, and Cochrane Library databases were searched to find studies reporting the incidence rates (IR) of melanoma and NMSC in patients with psoriasis and psoriatic arthritis treated with biologics or JAK inhibitors. Nineteen studies were included in the analysis with a total of 13,739 patients. The overall IR of melanoma was 0.08 (95% CI, 0.05–0.15) events per 100 PYs and the overall IR of NMSC was 0.45 (95% CI, 0.33–0.61) events per 100 PYs. The IRs of melanoma were comparable across patients treated with IL-17 inhibitors, IL-23 inhibitors, and JAK inhibitors, while the IRs of NMSC were higher in patients treated with JAK inhibitors than in those treated with biologics. Prospective, long-term cohort studies are required to reliably assess the risks associated with novel targeted therapies.

## 1. Introduction

Psoriasis is a prevalent chronic inflammatory skin disorder, affecting between 0.5% and 11.4% of adults, as well as 0% to 1.4% of children globally [1]. Notably, its occurrence appears to be on the rise in the last decades. Around 20% of patients with psoriasis develop psoriatic arthritis during their life course [2]. Both patients with psoriatic arthritis and patients with moderate-to-severe psoriasis have significantly impaired quality of life and often require long-term systemic treatment.

The risk of cancer in psoriatic patients has been a matter of debate for years. According to the latest meta-analysis, patients with psoriasis appear to have a slightly increased risk of developing cancer, particularly keratinocyte cancer, lymphomas, lung cancer, and bladder cancer [3]. Several factors have been attributed to the development of cancer in psoriatic patients. Firstly, well-known lifestyle factors, such as smoking and alcohol intake, which are common among individuals with psoriasis, could elevate their likelihood of developing cancer. Secondly, the relationship between the use of systemic treatment, such as phototherapy and immunosuppressive agents (methotrexate, cyclosporine A), has been reported, but the evidence is inconsistent [4,5,6].

One of the biggest breakthroughs in the treatment of psoriasis and psoriatic arthritis was the introduction of biologic agents (like TNF-α inhibitors, inhibitors of the IL-17 pathway, inhibitors of IL-23, and related cytokines) and Janus kinase (JAK) inhibitors. Owing to their immunomodulatory mechanism of action, there is concern that these biologics may increase the risk for infections and malignancies. According to the Psoriasis Longitudinal Assessment and Registry (PSOLAR), malignancy risk increased with long-term exposure to TNF-α inhibitors, but not with exposure to methotrexate or ustekinumab, anti-interleukin 12/23 antibody [6]. Skin malignancy is a matter of special concern in patients with psoriasis due to their association with sunlight exposure. To date, several studies sought to assess the risk of melanoma and non-melanoma skin cancer (NMSC) in patients treated with biologics and found that TNF-α inhibitors may increase this risk [7,8]. However, there is a paucity of data regarding the safety of newer biologic drugs (i.e., inhibitors of the IL-17 pathway, inhibitors of IL-23 and related cytokines, JAK inhibitors) in reference to skin cancer risk. Therefore, our aim was to assess what the risk of melanoma and non-melanoma skin cancer is in patients with psoriasis and psoriatic arthritis treated with novel targeted therapies.

## 2. Methods

This study was compliant with the guidelines of the Preferred Reporting Items for Systematic Reviews and Meta-Analyses (PRISMA) (Table S1) [9]. In accordance with The World Medical Association’s Declaration of Helsinki 2013, the research was registered in PROSPERO database (https://www.crd.york.ac.uk/PROSPERO, accessed on 28 July 2023) and “CRD42023439910” was assigned as its registration number. Ethical approval and patient consent were not required for a systematic review using meta-analysis.

### 2.1. Search Strategy

A search strategy was designed to identify all relevant randomized clinical trials and observational studies investigating cutaneous malignancy risk in patients with psoriasis and psoriatic arthritis treated with biologics and JAK inhibitors. A wide search using the MEDLINE/PubMed, EMBASE, Web of Science, and Cochrane Library databases was performed until 26 July 2023. The detailed search strategy is presented in Table S2. No publication date or language filters were applied. The reference lists of the eligible studies and relevant reviews were also manually scrutinized to find additional records.

### 2.2. Eligibility Assessment

Studies were included if they: (a) were RCTs, open-label studies, long-term extension studies or observational studies; (b) included adult patients suffering from psoriasis or psoriasis arthritis treated with biologics (IL-17 inhibitors: secukinumab, ixekizumab, brodalumab, bimekizumab; IL-12/23 inhibitor: ustekinumab; IL-23 inhibitors: risankizumab, guselkumab, tildrakizumab) or small molecules (JAK inhibitors: tofacitinib, upadacitinib, baricitinib); (c) reported IRs of melanoma and non-melanoma skin cancer presented as numbers of events per 100 PY or clearly reported number of skin cancer events with the total number of PY of exposure. We excluded narrative or systematic reviews and meta-analyses, book chapters, conference papers, and editorials. When multiple studies presented results from the same database or RCT, we only included the one with the most comprehensive data. Studies were excluded if they reported the total PY of follow-up instead of the total PY of exposure. Four independent reviewers (M.K., P.C., A.K., K.R.) selected the studies by screening the titles and abstracts found upon initial search. An eligibility assessment for the relevant full-text articles was performed by two independent authors (M.K. and A.K.). Each abstract and full-text article was assessed by at least two reviewers independently. In case of a lack of agreement between the reviewers, a consensus was reached by the whole review team.

### 2.3. Data Extraction and Risk of Bias Assessment

The following data were extracted from studies: title of article, name of the first author, year of publication, study design, database or clinical trial identifier, diagnosis, patient characteristics (sample size, age, sex), type(s) of biologic therapy, total PY of exposure, the use of concomitant therapy, outcomes of interest (IR of melanoma or non-melanoma skin cancer events per 100 PY). Both melanoma and melanoma in situ were considered ‘melanoma’ events, while basal cell carcinoma and squamous cell carcinoma were considered ‘NMSC’ events.

The risk of bias assessment was carried out by two independent authors (M.K. and A.K.). Each full-text article was assessed by two reviewers. Any discrepancy in the extracted data and risk of bias assessment was resolved by discussion with other team members. We used the Newcastle–Ottawa Quality Assessment Form for cohort studies [10] to assess the quality of the included nonrandomized articles, and the Cochrane risk of bias tool [11] for randomized controlled trials. The risk of bias assessment across studies is presented in Table S3.

### 2.4. Statistical Analysis

We evaluated skin cancer risk by calculating the IRs of skin neoplasm events per 100 PY. Analyses were stratified for specific skin cancer types, i.e., NMSCs and melanoma. Calculations were conducted using the metafor package in R (R Foundation for Statistical Computing) [12]. The results from individual studies were transformed using logarithmic or logit functions and analyzed through random effects meta-analysis using generalized linear mixed models with a Poisson distribution. The combined estimates were then converted back to their original scale. The Poisson model was previously shown to be flexible and has the advantage of avoiding zero-cell corrections [13]. Subgroup estimates were calculated individually for different drugs, groups of drugs, diseases, types of study, and follow-up duration. Heterogeneity between individual studies was quantified using Higgins I^2^ statistics [14]. Funnel plots were assessed for potential publication bias (Figure S1). If studies provided only summarized data without the ability to calculate specific numerical values, no synthesis of data was conducted. Sensitivity analyses were conducted by excluding point estimates from the meta-analysis to confirm that the overall risk estimates were not significantly influenced by individual studies.

## 3. Results

**Figure 1:**
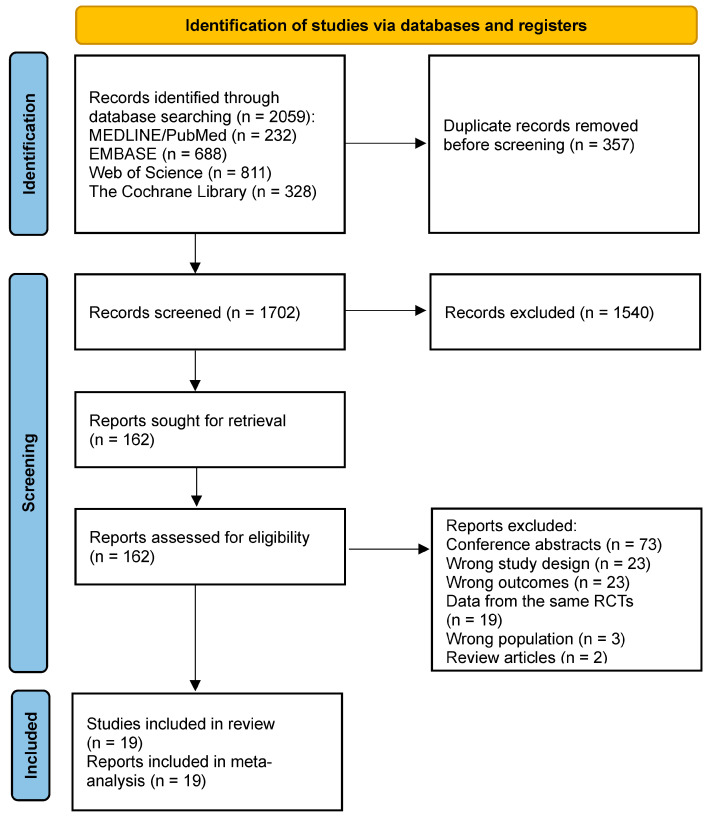
PRISMA flowchart outlining the study inclusion process.

The initial reference search yielded 2059 articles. Of these, 357 duplicates were removed and the remaining 1702 abstracts were screened. This produced 162 papers suitable for a full-text review. Reference screening of those studies did not provide additional records that met eligibility criteria. After full-text screening, 19 studies were subjected to extraction and quantitative synthesis (meta-analysis). The PRISMA study flow-chart outlining the study inclusion process is illustrated in Figure 1 [9]. The risk of bias assessment is presented in Table S3. In general, studies had high quality, although some studies were rated as having some concerns due to the lack of information about whether the outcome assessors were masked to the treatments when adjudicating cancer events.

### 3.1. Characteristics of the Included Studies

The main characteristics of the included studies are reported in Table 1. We included 1 cohort study [15] and 18 randomized controlled trials (RCT) [16,17,18,19,20,21,22,23,24,25,26,27,28,29,30,31,32,33] with a total of 13,739 patients. Twelve RCTs [17,18,21,22,24,25,26,28,30,31,32,33] included open-label or long-term extension periods. Eleven publications [15,18,19,20,21,22,23,24,27,28,30] reported skin malignancy risk in patients with psoriatic arthritis and 8 [16,17,25,26,29,31,32,33] in patients with psoriasis. Our analysis included one study focusing on IL-12/23 inhibitor ustekinumab [15], eight studies focusing on IL-23 inhibitors (guselkumab [17,20], tildrakizumab [33], risankizumab [16,24,29,30,32]), seven studies focusing on IL-17 inhibitors (bimekizumab [21], ixekizumab [22,26,28], brodalumab [25], secukinumab [23,27]), and three studies focusing on JAK inhibitors (upadacitinib [19], tofacitinib [18,31]). The duration of follow-up for melanoma and NMSC occurrence ranged from 24 weeks to 5 years. Nine of the studies had a follow-up duration of ≤2.5 years and 10 of the studies >2.5 years.

### 3.2. Melanoma Risk

**Figure 2:**
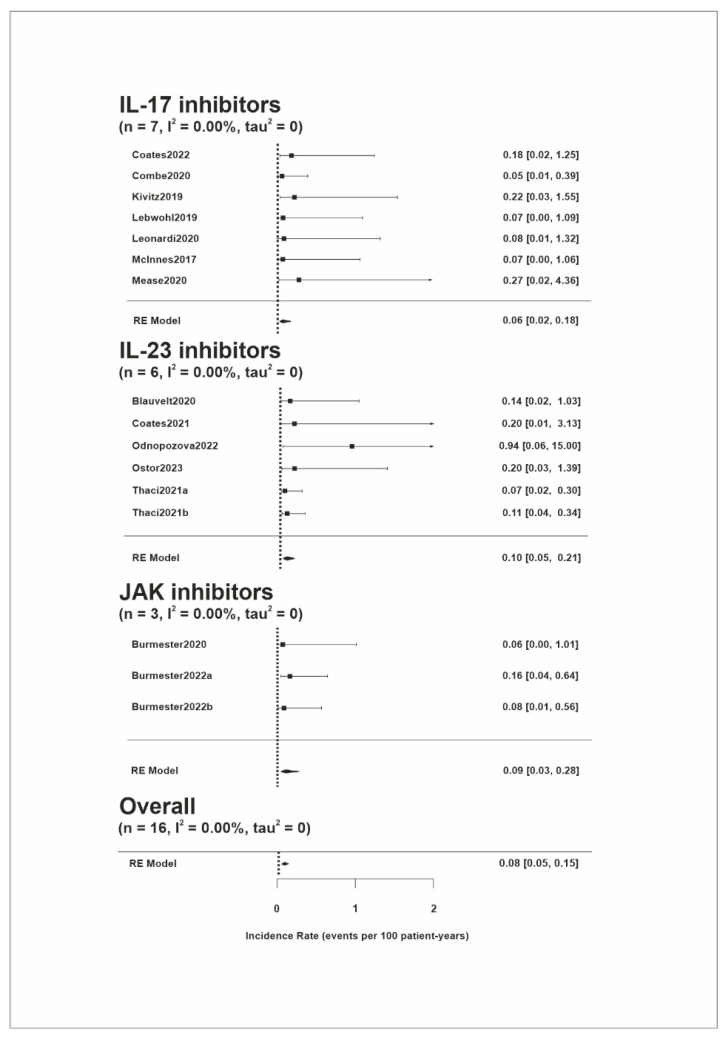
Forest plot of the estimated melanoma incidence per 100 patient years with 95% confidence intervals according to the specific treatment [16,18,19,20,21,22,23,25,26,27,28,29,30,33]. The boxes represent the point estimates, with horizontal lines indicating 95% confidence intervals. Diamonds represent pooled estimates with tips of the diamonds indicating 95% confidence intervals. JAK, Janus kinase; RE, random effects model.

The incidence rates (IRs) of melanoma are presented in Figure 2. Melanoma risk was reported in 14 studies. The overall IR was 0.08 (95% CI, 0.05–0.15) events per 100 patient years (PYs). There was no heterogeneity between the studies (Tau^2^ = 0.0; I^2^ = 0.0%). Patients treated with IL-17 inhibitors had a pooled IR of 0.06 (95% CI, 0.02–0.18) melanoma events per 100 PYs, patients treated with IL-23 inhibitors had 0.10 (95% CI, 0.05–0.21) events per 100 PYs, and patients treated with JAK inhibitors had 0.09 (95% CI, 0.03–0.28) events per 100 PYs. The fact that all studies reporting malignancy risk in patients with psoriatic arthritis allowed for the concomitant use of other immunosuppressive drugs (i.e., methotrexate); hence, we conducted subgroup analysis according to the disease. The IRs of melanoma in this subgroup were 0.09 (95% CI, 0.04–0.19) events per 100 PYs and 0.08 (95% CI, 0.04–0.18) events per 100 PYs in patients with psoriatic arthritis and psoriasis, respectively (Figure S2). The analysis of only those studies with a longer follow-up period (>2.5 years) is shown in Figure S3. Sensitivity analysis is presented in Table S4.

### 3.3. NMSC Risk

**Figure 3:**
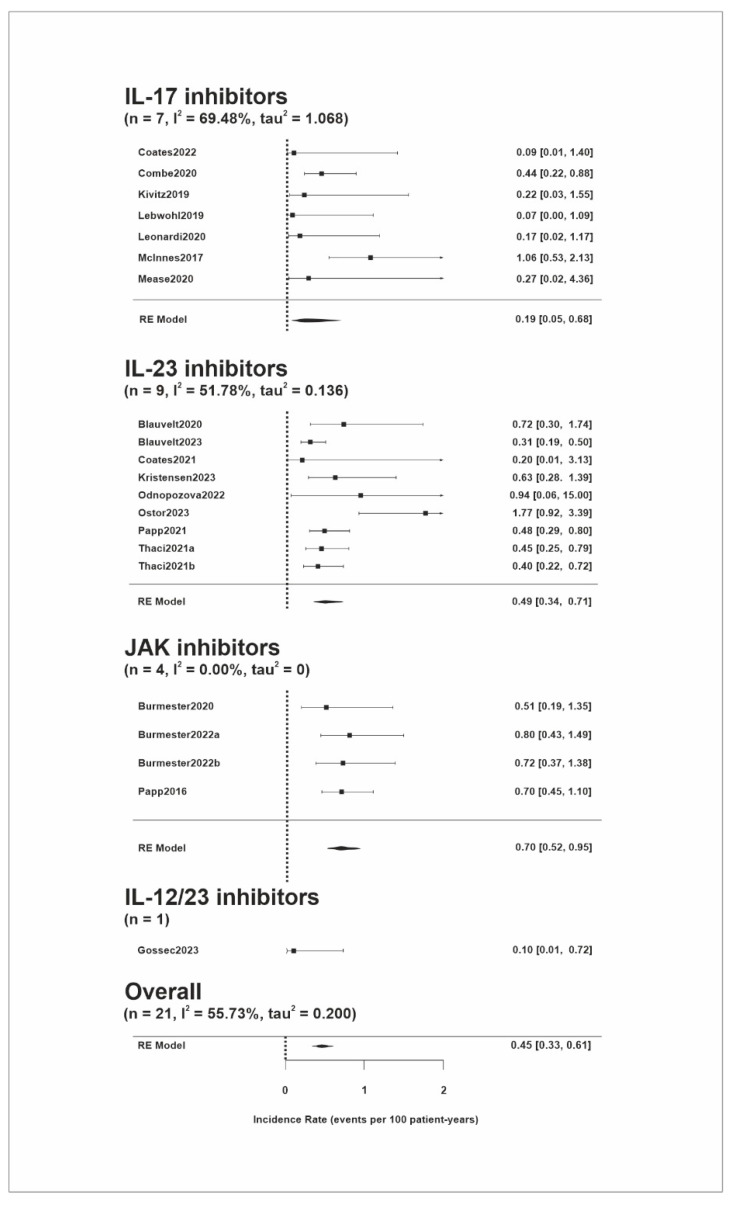
Forest plot of the estimated non-melanoma skin cancer incidence per 100 patient years with 95% confidence intervals according to the specific treatment [15,16,17,18,19,20,21,22,23,24,25,26,27,28,29,30,31,32,33]. Boxes indicate point estimates, with horizontal lines indicating 95% confidence intervals. Diamonds indicate pooled estimates with tips of the diamonds indicating 95% confidence intervals. JAK, Janus kinase; RE, random effects model.

The IRs of NMSC are presented in Figure 3. The NMSC risk was presented in 19 studies. The overall estimate of the IR was 0.45 (95% CI, 0.33–0.61) events per 100 PYs. The heterogeneity between the studies was moderate (Tau^2^ = 0.2; I^2^ = 55.7%). In a subgroup analysis, patients treated with IL-17 inhibitors had a pooled IR of 0.19 (95% CI, 0.05–0.68) NMSC events per 100 PYs, patients treated with IL-23 inhibitors had 0.49 (95% CI, 0.34–0.71) events per 100 PYs, and patients treated with JAK inhibitors had 0.70 (95% CI, 0.52–0.95) events per 100 PYs. The IRs of NMSC were 0.47 (95% CI, 0.28–0.81) events per 100 PYs in patients with psoriatic arthritis and 0.42 (95% CI, 0.32–0.55) events per 100 PYs in patients with psoriasis (Figure S2). The analyses of studies with a longer follow-up period (>2.5 years) and of randomized controlled trials only are shown in Figure S3 and Figure S4, respectively. Sensitivity analysis is presented in Table S4.

## 4. Discussion

Our systematic review and meta-analysis aimed to analyze the risk of melanoma and non-melanoma skin cancer in patients with psoriasis and psoriatic arthritis treated with targeted therapies. To the best of our knowledge, this is the first analysis to summarize the risk of skin malignancy in patients undergoing treatment with IL-17 and IL-23 inhibitors. Up until now, the majority of research concentrated on TNF-α inhibitors and, to a lesser extent, ustekinumab and JAK inhibitors. In a comparable research primarily involving patients treated with TNF-α inhibitors, the IRs of melanoma and NMSC were 6.1 and 124.5 per 10,000 PY, respectively [34]. The melanoma risk was similar to that in our study (0.06 vs. 0.08 per 100 PY), though the risk of non-melanoma skin cancer was somewhat higher (1.25 vs. 0.45 per 100 PY). Unlike our study, the mentioned meta-analysis did not conduct subgroup analyses based on specific drug groups or diseases. According to the Centers for Disease Control and Prevention, in 2018, the IR of melanoma in general population was 0.02 per 100 persons [35]. Additionally, the age-standardized incidence rate of NMSC was reported at 0.08 per 100 in 2019 [36]. To date, two meta-analyses have assessed the risk of melanoma and NMSC in patients treated with biologics compared to those who had not received biologic therapy. The results indicate that there is a clinically important increase in skin malignancy risk; however, these findings predominantly pertain to patients treated with TNF-α inhibitors [7,8].

There seems to be a fairly evident link between psoriasis and skin cancers, which may be attributed to the usage of systemic therapies and environmental factors. Ultraviolet radiation (UVR) and immunosuppression are key factors playing a role in the development of melanoma and NMSC. Given that melanoma is regarded as one of the most aggressive types of cancer, it should be particularly taken into consideration when determining treatment strategy. The etiology and pathogenesis of melanoma involve complex interactions between genetic factors and environmental exposures, such as UVR from natural or artificial sources. UVR induces carcinogenesis in two independent pathogenetic pathways: a melanin-independent pathway related to direct UVB-induced DNA damage and a UVA-initiated, pigment-dependent pathway associated with indirect oxidative damage to the DNA in melanocytes [37]. Melanogenesis, along with its highly reactive intermediates, shows cytotoxic, genotoxic, and mutagenic properties, which can contribute to melanoma progression. On the other hand, there is some evidence that the presence of melanin can inhibit the formation of melanoma metastases. Both of the above-mentioned phenomena are referred to as the Yin and Yang effects [38,39]. It is thought that immunosuppression can induce melanocyte-stimulating hormone or melanoma growth-stimulatory activity, which, along with genetic factors, can accelerate the progression of melanoma and is a matter of concern in the context of immunosuppressive therapy [40].

The recent introduction of systemic biologic drugs (TNF-α inhibitors, the anti-IL12/23 ustekinumab, the IL-17/IL-17 receptor antagonists, and the anti-IL-23 agents) alongside chemically synthesized medications (JAK inhibitors) has revolutionized the treatment of psoriasis and psoriatic arthritis, offering more targeted therapeutic options. Given their immunosuppressive nature, it is crucial to continually update our knowledge regarding the safety of their use. Biologic agents, which function by targeting the IL-23/IL-17/Th17 axis, are characterized by very high efficacy in the treatment of psoriasis and psoriatic arthritis. Ustekinumab, an anti-IL-12p40 monoclonal antibody, was one of the first extensively utilized antibody-targeting IL-12/23s. The IL-12p40 subunit is a component of both IL-12 and IL-23 [41]. This fact has led researchers to innovate and create further drugs that interact with IL-23 and IL-17. Biologic agents targeting IL-17 and IL-23 are typically well-tolerated and safe, although there is a higher incidence of adverse events when compared to the placebo. According to Loft et al. [42], patients receiving IL-23 inhibitors have a lower incidence of any adverse events compared to those treated with IL-17 inhibitors. This difference could potentially be explained by an increased rate of infections within the latter group. The risk of malignancy was low in both groups [42]. In our research, individuals treated with IL-17 inhibitors had a lower IR of either melanoma or NMSC than those on IL-23 inhibitors (0.06 vs. 0.10 per 100 PYs and 0.19 vs. 0.49 per 100 PYs, respectively).

JAK inhibitors target Janus kinases, intracellular non-receptor tyrosine kinases that play a crucial role in translating extracellular signals into various cellular responses. They constitute a widely used group of medications in immune-mediated inflammatory diseases such as psoriatic arthritis, ankylosing spondylitis, ulcerative colitis and polyarticular juvenile idiopathic arthritis. Due to the potentially increased risk of cardiovascular side effects and cancer, several meta-analyses have been conducted to investigate the risks associated with its use [43,44,45]. Notably, patients treated with JAK inhibitors were found to have an increased risk of cancer compared to those receiving TNF-α inhibitors [43,44]. In our study, patients who received JAK inhibitors had similar Irs of melanoma to those treated with biologics, yet they demonstrated a greater risk of NMSC.

It is crucial to take into account some limitations when interpreting the results of this meta-analysis. The number of included studies is relatively small, partly due to the established inclusion criteria requiring the expression of IR as events per 100 PY. Another limit identified in our meta-analysis is the follow-up time. Skin malignancies have a long development and growth time; thus, the follow-up duration in the included studies might have been insufficient and resulted in an underestimation of the associated risks. However, this limitation is unrelated to the study concept itself and results directly for available studies. Due to a very limited number of studies comparing the risk of skin cancer among different targeted drugs over a longer time, we were unable to perform a comparative analysis of safety for particular drugs. Moreover, we were unable to compare the safety of the analyzed drugs with a healthy population or with biologicnaïvee patients, as was done by Esse et al. [7] or Liu et al. [8]. Another significant weakness of the analysis was the absence of adjustment for established risk factors for NMSC and melanoma, such as exposure to UVR or the prior usage of immunosuppressive treatment. Unfortunately, these parameters were infrequently given in the original studies and—at least exposure to UV/sun—are difficult to objectively measure. Moreover, RCTs including patients with psoriatic arthritis allowed for the concomitant use of targeted drugs and methotrexate, which could have influenced the risk of skin cancers.

The major strength of this study Is the inclusion of a high-quality cohort study and RCTs, offering the most current and reliable evidence based on four large biomedical databases. Furthermore, we used a well-defined protocol with explicit criteria for inclusion and exclusion. All included studies scored relatively high on the Cochrane risk of bias tool or the Newcastle–Ottawa Quality Assessment Form for Cohort Studies. Previous studies have predominantly focused on the risk of melanoma and NMSC in patients treated with TNF-α inhibitors. Here, we reported the best available evidence on the safety of newer biologics and JAK inhibitors.

## 5. Conclusions

In this study, we present a comprehensive review and meta-analysis of skin cancer risk associated with novel biologic therapies. Overall, the risk of melanoma and NMSC is higher in patients treated with biologics for psoriasis as opposed to the general population. The IRs of melanoma seem to be comparable across patients treated with newer drugs (IL-17 inhibitors, IL-23 inhibitors, JAK inhibitors), and they similarly correspond to the previously published findings concerning patients receiving TNF-α inhibitors. The risk of NMSC is the lowest in the subgroup of patients treated with IL-17 inhibitors and ustekinumab and the highest in patients treated with JAK inhibitors. Prospective, long-term cohort studies using an active or placebo comparator are required to reliably assess the risks associated with novel targeted therapies. Furthermore, an assessment of the safety of targeted therapies in populations at increased risk of skin cancer is recommended, along with the establishment of guidelines for their use and monitoring in these specific populations.

## Figures and Tables

**Table 1:** Characteristics of the included studies.

Source	Identifier	Study Design	Disease	Study Drug	Drug Dose	No. of Participants	Total Patient Years of Exposure	Age, Mean (SD)	Male, *n* (%)	Melanoma Incidence Rates per 100 Patient Years	NMSC Incidence Rates per 100 Patient Years
Observational studies											
Gossec, 2023 [15]	PsaBIO study (NCT02627768)	Multinational, prospective, real-world, observational study	PSA	Ustekinumab (IL-12/23 inhibitor)	45 mg/90 mg	494	991.3	NA	NA	NA	0.1
Randomized clinical trials											
Blauvelt, 2020 [16]	IMMhance (NCT02672852)	Phase 3, multinational, double-blind placebo-controlled trial	PSO	Risankizumab (IL-23 inhibitor)	150 mg	500	690.1	NA	NA	0.14	0.72
Blauvelt, 2023 [17]	VOYAGE 1 (NCT02207231), VOYAGE 2 (NCT02207244)	Randomized, double-blind, phase 3 studies with OLE	PSO	Guselkumab (IL-23 inhibitor)	100 mg	1221	>5200	NA	NA	NA	0.31 (0.17–0.5)
Burmester, 2020 [18]	OPAL Broaden (NCT01877668), OPAL Beyond (NCT01882439), OPAL Balance (NCT01976364)	Double-blind, placebo-controlled, parallel-group studies with LTE	PSA	Tofacitinib (JAK inhibitor)	5 mg/10 mg	783	789	48.7 (12.0)	355 (45)	0	0.5 (0.1–1.3)
Burmester, 2022a [19]	SELECT-PsA 1 (NCT03104400), SELECT-PsA 2 (NCT03104374)	Randomized, placebo-controlled phase 3 trials	PSA	Upadacitinib (JAK inhibitor)	15 mg	907	1247.2	51.5 (12.1)	429 (47)	0.16	0.8 (0.4–1.5)
Burmester, 2022b [19]					30 mg	921	1257.4	51.4 (12.3)	417 (45)	0.08	0.8 (0.4–1.5)
Coates, 2021 [20]	COSMOS (NCT03796858)	Phase IIIb, randomized, double-blind study	PSA	Guselkumab (IL-23 inhibitor)	100 mg	279	255.4	NA	NA	0	0
Coates, 2022 [21]	BE ACTIVE (NCT02969525)	Randomized, double-blind, placebo-controlled study with OLE	PSA	Bimekizumab (IL-17 inhibitor)	160 mg/320 mg	206	570.1	49.3 (12.4)	105 (51)	0.2	0
Combe, 2020 [22]	SPIRIT-P1 (NCT01695239), SPIRIT-P2 (NCT02349295), SPIRIT-P3 (NCT02584855)	Phase 3 randomized, double-blind, placebo-controlled, parallel-group studies with LTE; phase 3 study with an open-label period followed by a randomized double-blind withdrawal period	PSA	Ixekizumab (IL-17 inhibitor)	160 mg → 80 mg	1118	1822.2	49.5 (11.9)	517 (46.2)	0.05	0.4 (0.1–3.0)
Kivitz, 2019 [23]	FUTURE 4 (NCT02294227)	Randomized, Double-blind, Placebo-controlled Multicenter Study	PSA	Secukinumab (IL-17 inhibitor)	150 mg	334	458.4	NA	NA	0.22	0.22
Kristensen, 2023 [24]	KEEPsAKE 1 (NCT03675308)	Phase 3, double-blind, placebo-controlled study with OLE	PSA	Risankizumab (IL-23 inhibitor)	150 mg	946	958.1	NA	NA	NA	0.6
Lebwohl, 2019 [25]	NCT00975637, NCT01101100	Phase II, double-blind, placebo-controlled, dose-ranging clinical trial with OLE	PSO	Brodalumab (IL-17 inhibitor)	210 mg	181	731.7	42.7 (12.2)	117 (65)	0	0
Leonardi, 2020 [26]	UNCOVER-1 (NCT01474512), UNCOVER-2 (NCT01597245)	Multicenter, randomized, double-blinded, placebo-controlled, phase-3 clinical trials with LTE	PSO	Ixekizumab (IL-17 inhibitor)	160 mg → 80 mg	206	604.3	Median (range) 43.0 (18–77)	140 (68)	0	0.17
McInnes, 2017 [27]	FUTURE 2 (NCT01752634)	Multicenter, randomized, double-blind, parallel-group, placebo-controlled study	PSA	Secukinumab (IL-17 inhibitor)	75 mg/150 mg/300 mg	387	751.3	NA	NA	0	1.06
Mease, 2020 [28]	SPIRIT-H2H (NCT03151551)	Phase IIIb/IV, multicenter, randomized, open-label, blinded-assessor, parallel-group study	PSA	Ixekizumab (IL-17 inhibitor)	160 mg→80 mg	283	183.5	47.5 (12.0)	162 (57)	0	0
Odnopozova, 2022 [29]	IMMPress (NCT03518047)	Phase 3, randomized, double-blind, placebo-controlled study	PSO	Risankizumab (IL-23 inhibitor)	150 mg	50	53.3	44.6 (12.9)	27 (54)	0	0
Östor, 2023 [30]	KEEPsAKE 2 (NCT03671148)	Double-blind, placebo-controlled study with OLE	PSA	Risankizumab (IL-23 inhibitor)	150 mg	419	509.7	NA	NA	0.2	1.76
Papp, 2016 [31]	OPT Pivotal 1 (NCT01276639), OPT Pivotal 2 (NCT01309737), NCT01163253	Phase III, multisite, randomized, double-blind clinical studies with LTE	PSO	Tofacitinib (JAK inhibitor)	5 mg/10 mg	1807	2704.8	NA	NA	NA	0.71 (0.43–1.1)
Papp, 2021 [32]	LIMMitless (NCT03047395)	Phase III OLE study	PSO	Risankizumab (IL-23 inhibitor)	150 mg	897	3106.2	46.9 (22.4)	633 (71)	NA	0
Thaci, 2021a [33]	reSURFACE 1 (NCT01722331), reSURFACE 2 (NCT01729754)	Randomized, double-blind, placebo-controlled, parallel-group phase III trials with LTE	PSO	Tildrakizumab (IL-23 inhibitor)	100 mg	872	2688.4	NA	NA	0.1 (0.0–0.3)	0.4 (0.2–0.8)
Thaci, 2021b [33]					200 mg	928	2753.5	NA	NA	0.1 (0.0–0.3)	0.4 (0.2–0.7)

Abbreviations: SD, standard deviation; NMSC, non-melanoma skin cancer; PSA, psoriatic arthritis; PSO, psoriasis; NA, not available; OLE, open-label extension; LTE, long-term extension; JAK, Janus kinase.

## Data Availability

Data is available from the authors upon reasonable request.

## Supplementary Materials

The following supporting information can be downloaded at: https://www.mdpi.com/article/10.3390/ph17010014/s1, Figure S1: Funnel plot for the inspection of publication bias according to the disease. Figure S2: Forest plot of the estimated melanoma and non-melanoma skin cancer incidence per 100 patient years with 95% confidence intervals according to the disease. Figure S3: Forest plot of the estimated melanoma and non-melanoma skin cancer incidence per 100 patient years with 95% confidence intervals: subgroup analysis of studies with a follow-up period longer than 2.5 years. Figure S4: Forest plot of the estimated non-melanoma skin cancer incidence per 100 patient years with 95% confidence intervals: subgroup analysis of randomized controlled trials only. Table S1: PRISMA 2020 Checklist. Table S2: Search strategy. Table S3: Summary of the risk of bias assessment. Table S4: Leave-one-out summary.
