# Supplementary material for: Risk of Melanoma and Non-Melanoma Skin Cancer in Patients with Psoriasis and Psoriatic Arthritis Treated with Targeted Therapies: A Systematic Review and Meta-Analysis
Source: Pharmaceuticals (Basel). 2023 Dec 21;17(1):14. doi: 10.3390/ph17010014 (PMC10820691; doi:10.3390/ph17010014)

**Figure S1**: Funnel plot for the inspection of publication bias according to the disease. Each point on the graph represents the standard error and logarithmic incidence rate from an individual study. The white triangle indicates the area where 95% of the data points would lie in the absence of a publication bias.

1. Melanoma


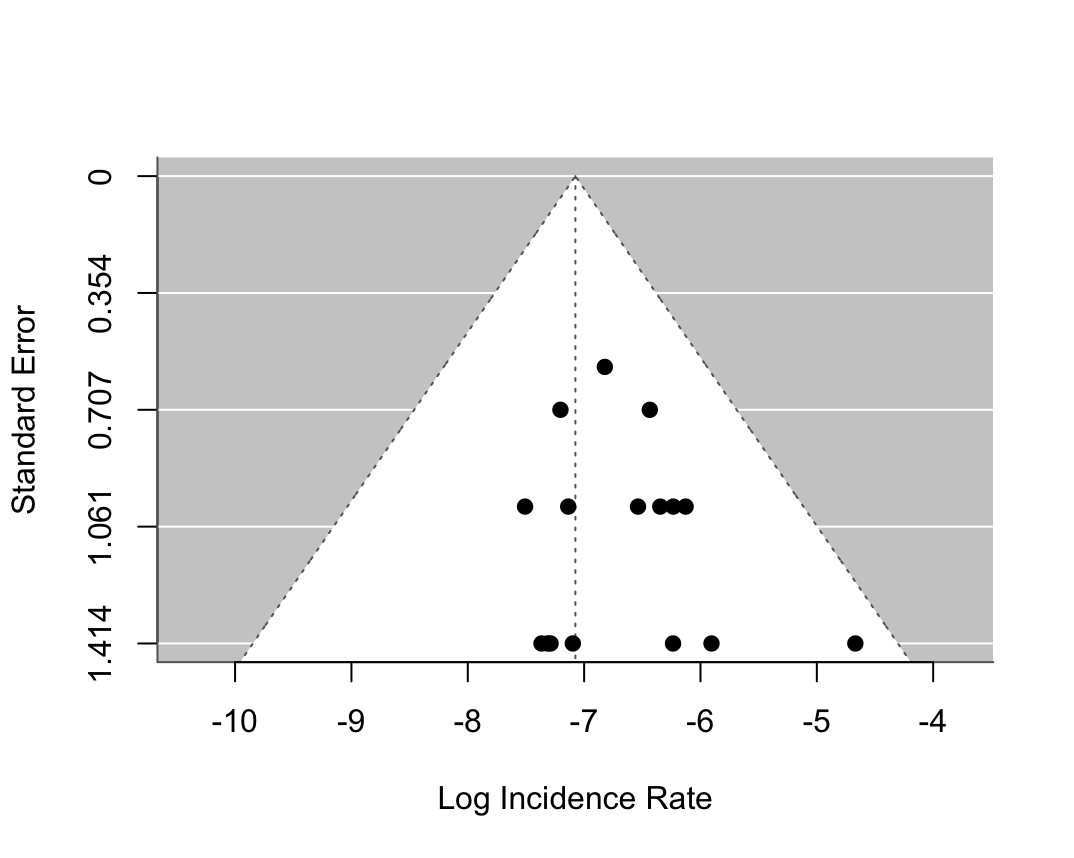


1. Non-melanoma skin cancer


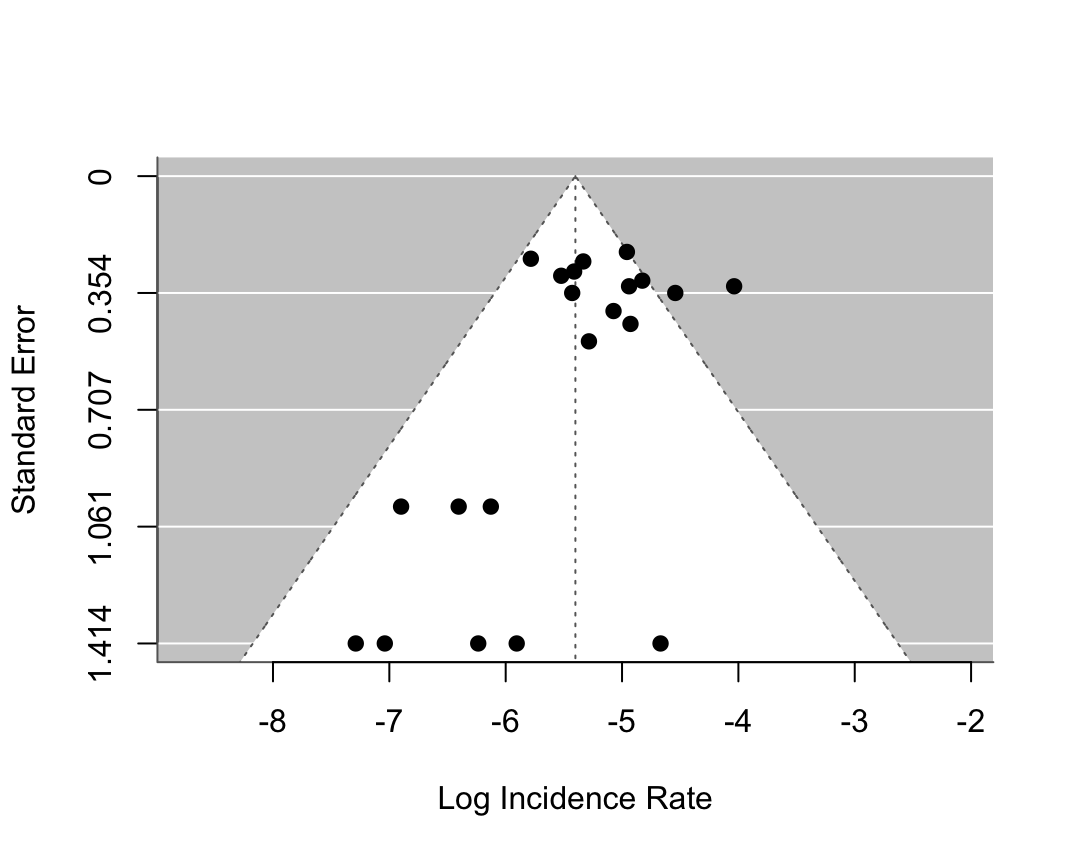

Supplement: Supplementary file 1 [file pharmaceuticals-17-00014-s001.zip › Figure S1.docx]
