# Supplementary material for: Risk of Melanoma and Non-Melanoma Skin Cancer in Patients with Psoriasis and Psoriatic Arthritis Treated with Targeted Therapies: A Systematic Review and Meta-Analysis
Source: Pharmaceuticals (Basel). 2023 Dec 21;17(1):14. doi: 10.3390/ph17010014 (PMC10820691; doi:10.3390/ph17010014)

**Figure S3**: Forest plot of the estimated melanoma and non-melanoma skin cancer incidence per 100 patient-years with 95% confidence intervals – subgroup analysis of studies with follow-up period longer than 2.5 years. Boxes represent point estimates, with horizontal lines indicating 95% confidence intervals. Diamonds represent pooled estimates with tips of the diamonds indicating 95% confidence intervals. RE, random effects model.


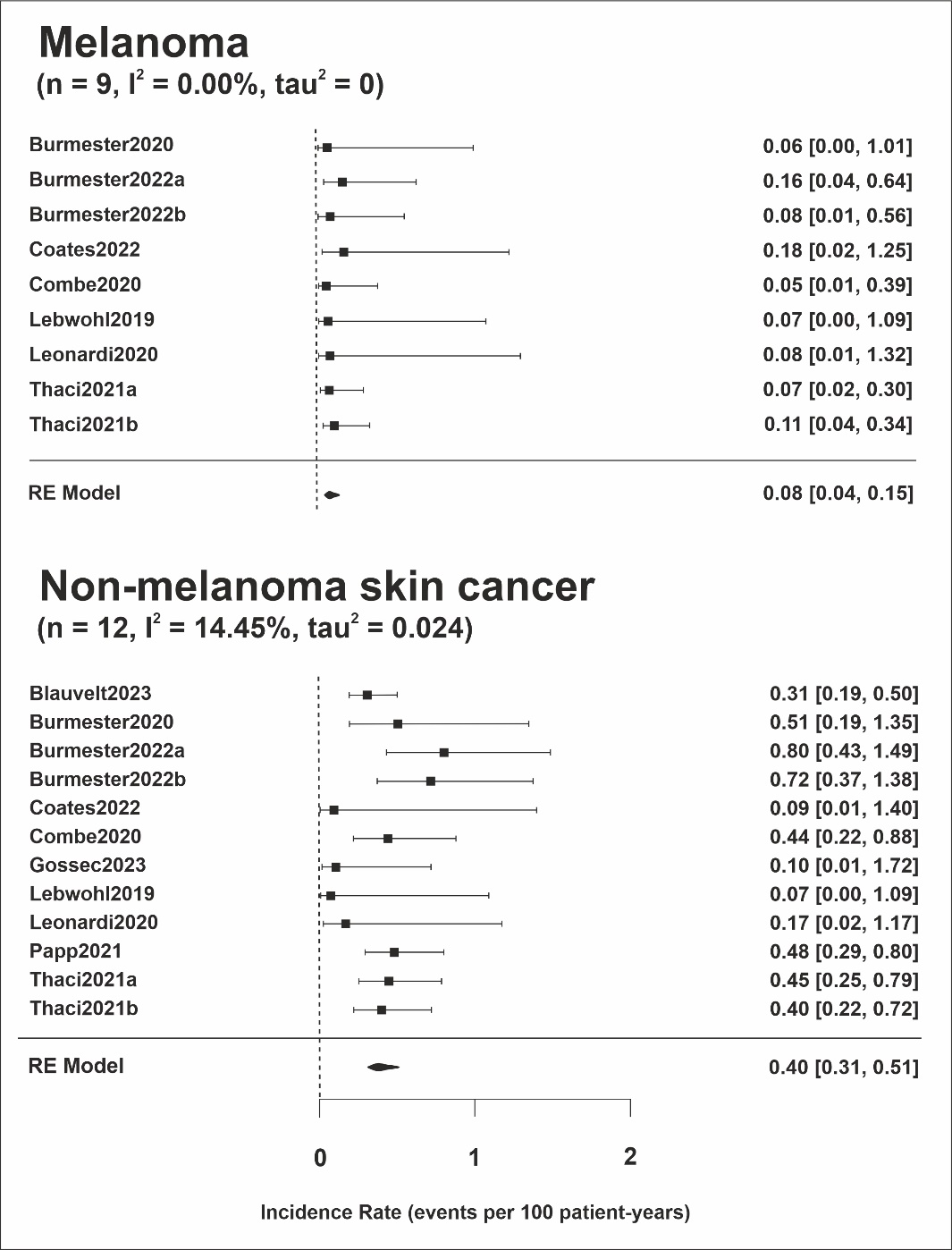

Supplement: Supplementary file 1 [file pharmaceuticals-17-00014-s001.zip › Figure S3.docx]
