# Supplementary material for: Risk of Melanoma and Non-Melanoma Skin Cancer in Patients with Psoriasis and Psoriatic Arthritis Treated with Targeted Therapies: A Systematic Review and Meta-Analysis
Source: Pharmaceuticals (Basel). 2023 Dec 21;17(1):14. doi: 10.3390/ph17010014 (PMC10820691; doi:10.3390/ph17010014)
Supplement: Supplementary file 1 [file pharmaceuticals-17-00014-s001.zip › Table S2.docx]

| MEDLINE/PubMed | | |
| --- | --- | --- |
| #1 | ((((((psoriasis[MeSH Terms]) OR (psoriasis)) OR (psoria*[Title/Abstract])) OR (arthritic psoriasis[MeSH Terms])) OR (arthritis, psoriatic[MeSH Terms])) OR (arthritic psoriasis)) OR (arthritic psoriasis[Title/Abstract]) | 67085 |
| #2 | ((((((((((((((etanercept) OR (Infliximab)) OR (Adalimumab)) OR (Ustekinumab)) OR (Secukinumab)) OR (Ixekizumab)) OR (Brodalumab)) OR (Guselkumab)) OR (Tildrakizumab)) OR (Risankizumab)) OR (certolizumab)) OR (bimekizumab)) OR (baricitinib)) OR (tofacitinib)) OR (upadacitinib) | 36505 |
| #3 | #1 AND #2 | 8378 |
| #4 | (((((((((skin neoplasm[MeSH Terms]) OR (skin neoplasm*[Title/Abstract])) OR (skin cancer[MeSH Terms])) OR (skin cancer*)) OR (skin tumor[Title/Abstract])) OR (skin tumour*[Title/Abstract])) OR (skin malignanc*[Title/Abstract])) OR ((carcinoma, squamous cell[MeSH Terms]) OR ("squamous cell carcinoma"))) OR ((carcinoma, basal cell[MeSH Terms]) OR ("basal cell carcinoma"))) OR ((melanoma [MeSH Terms]) OR (melanoma)) | 478831 |
| #5 | #3 AND #4 | 232 |

Table S2: Search strategy

| EMBASE | | |
| --- | --- | --- |
| #1 | 'psoriasis'/exp OR 'psoriasis' | 123305 |
| #2 | 'psoriatic arthritis'/exp OR 'psoriatic arthritis' | 33114 |
| #3 | #1 AND #2 | 31972 |
| #4 | 'etanercept'/exp OR 'etanercept' OR 'infliximab'/exp OR 'infliximab' OR 'adalimumab'/exp OR 'adalimumab' OR 'ustekinumab'/exp OR 'ustekinumab' OR 'secukinumab'/exp OR 'secukinumab' OR 'ixekizumab'/exp OR 'ixekizumab' OR 'brodalumab'/exp OR 'brodalumab' OR 'guselkumab'/exp OR 'guselkumab' OR 'tildrakizumab'/exp OR 'tildrakizumab' OR 'risankizumab'/exp OR 'risankizumab' OR 'certolizumab'/exp OR 'certolizumab' OR 'bimekizumab'/exp  OR 'bimekizumab' OR 'baricitinib'/exp  OR 'baricitinib' OR 'tofacitinib'/exp  OR 'tofacitinib' OR 'upadacitinib'/exp  OR 'upadacitinib' | 114075 |
| #5 | #3 AND #4 | 11677 |
| #6 | 'skin tumor'/exp OR 'skin tumor' OR 'skin cancer'/exp OR 'skin cancer' OR 'skin tumour'/exp OR 'skin tumour' OR 'skin neoplasm'/exp OR 'skin neoplasm' OR 'squamous cell carcinoma'/exp OR 'squamous cell carcinoma' OR 'basal cell carcinoma'/exp OR 'basal cell carcinoma' OR 'melanoma'/exp OR 'melanoma' | 684573 |
| #7 | #5 AND #6 | 688 |

| Web of Science | | |
| --- | --- | --- |
| #1 | (TS=(psoriasis)) OR TS=(psoriatic arthritis) | 77747 |
| #2 | ((((((((((((((TS=(etanercept)) OR TS=(infliximab)) OR TS=(adalimumab)) OR TS=(ustekinumab)) OR TS=(secukinumab)) OR TS=(ixekizumab)) OR TS=(brodalumab)) OR TS=(guselkumab)) OR TS=(tildrakizumab)) OR TS=(risankizumab)) OR TS=(certolizumab)) OR TS=(bimekizumab)) OR TS=(baricitinib)) OR TS=(tofacitinib)) OR TS=(upadacitinib) | 59186 |
| #3 | ((((((TS=(skin tumor)) OR TS=(skin tumour)) OR TS=(skin cancer)) OR TS=(skin neoplasm)) OR TS=(squamous cell carcinoma)) OR TS=(basal cell carcinoma)) OR TS=(melanoma) | 472512 |
| #4 | #1 AND #2 AND #3 | 811 |

| The Cochrane Library | | |
| --- | --- | --- |
| #1 | (psoriasis):ti,ab,kw OR (psoriatic arthritis):ti,ab,kw | 10880 |
| #2 | (etanercept):ti,ab,kw OR (infliximab):ti,ab,kw OR (adalimumab):ti,ab,kw OR (ustekinumab):ti,ab,kw OR (secukinumab):ti,ab,kw OR (ixekizumab):ti,ab,kw OR (brodalumab):ti,ab,kw OR (guselkumab):ti,ab,kw OR (tildrakizumab):ti,ab,kw OR (risankizumab):ti,ab,kw OR (certolizumab):ti,ab,kw OR (bimekizumab):ti,ab,kw OR (baricitinib):ti,ab,kw OR (tofacitinib):ti,ab,kw OR (upadacitinib):ti,ab,kw | 12843 |
| #3 | (skin tumor):ti,ab,kw OR (skin tumour):ti,ab,kw OR (skin cancer):ti,ab,kw OR (skin neoplasm):ti,ab,kw OR (squamous cell carcinoma):ti,ab,kw OR (basal cell carcinoma):ti,ab,kw OR (melanoma):ti,ab,kw | 24453 |
| #4 | #1 AND #2 AND #3 | 328 |
