# Supplementary material for: Risk of Melanoma and Non-Melanoma Skin Cancer in Patients with Psoriasis and Psoriatic Arthritis Treated with Targeted Therapies: A Systematic Review and Meta-Analysis
Source: Pharmaceuticals (Basel). 2023 Dec 21;17(1):14. doi: 10.3390/ph17010014 (PMC10820691; doi:10.3390/ph17010014)
Supplement: Supplementary file 1 [file pharmaceuticals-17-00014-s001.zip › Table S3.docx]

Table S3: Summary of Risk of Bias Assessment

| Author | Year | Selection Bias Assessment | | | | Comparability | Outcome | | | Total* |
| --- | --- | --- | --- | --- | --- | --- | --- | --- | --- | --- |
|  |  | Representativeness of the exposed cohort | Selection of the non-exposed cohort | Ascertainment of exposure | Demonstration that outcome of interest was not present at start of study | Comparability of cohorts on the basis of the design or analysis controlled for confounders | Assessment of outcome | Was follow-up long enough for outcomes to occur | Adequacy of follow-up of cohorts |  |
| Gossec | 2023 | 1 | 0 | 1 | 1 | 2 | 1 | 1 | 1 | 8 |
| *maximum score is 9 | | | | | | | | | | |

Cohort studies^a^

Randomized controlled trials^b^

| Author | Year | Risk of bias arising from the randomization process | Risk of bias due to deviations from the intended interventions | Missing outcome data | Risk of bias in measurement of the outcome | Risk of bias in selection of the reported result | Overall risk of bias |
| --- | --- | --- | --- | --- | --- | --- | --- |
| Thaci | 2021 | Low | Low | Low | Some concerns | Low | Low |
| Kristensen | 2023 | Low | Low | Low | Some concerns | Low | Low |
| Ostor | 2023 | Low | Low | Low | Some concerns | Low | Low |
| Kivitz | 2019 | Low | Low | Low | Some concerns | Low | Low |
| McInnes | 2017 | Low | Low | Low | Some concerns | Low | Low |
| Coates | 2022 | Low | Low | Low | Low | Low | Low |
| Papp | 2016 | Low | Low | Low | Some concerns | Low | Low |
| Burmester | 2022 | Low | Low | Some concerns | Some concerns | Low | Low |
| Burmester | 2020 | Low | Low | Some concerns | Some concerns | Low | Low |
| Coates | 2022 | Low | Low | Low | Some concerns | Low | Low |
| Odnopozova | 2022 | Low | Some concerns | Low | Some concerns | Low | Low |
| Blauvelt | 2020 | Low | Low | Low | Low | Low | Low |
| Papp** | 2021 | NA | NA | NA | NA | NA | NA |
| Blauvelt | 2023 | Low | Low | Low | Some concerns | Low | Low |
| Langley | 2015 | Low | Low | Low | Some concerns | Low | Low |
| Kimball | 2013 | Low | Low | Low | Low | Low | Low |
| Combe | 2020 | Low | Low | Low | Some concerns | Low | Low |
| Mease | 2020 | Low | Some concerns | Low | Some concerns | Low | Low |
| Leonardi | 2021 | Low | Low | Low | Low | Low | Low |
| Lebwohl | 2019 | Low | Low | Low | Some concerns | Low | Low |
| **open-label extension study without control arm | | | | | | | |

^a^Risk of bias assessed by the Newcastle-Ottawa Scale. In each domain, 1 corresponds to low risk of bias and 0 corresponds to high risk of bias. Studies with a total score (namely the sum score of all domains) of 7 or more points were considered high-quality studies.

^b^Risk of bias assessed by the Cochrane Risk of Bias Tool (RoB, version 2.0).
