# Supplementary material for: Risk of Melanoma and Non-Melanoma Skin Cancer in Patients with Psoriasis and Psoriatic Arthritis Treated with Targeted Therapies: A Systematic Review and Meta-Analysis
Source: Pharmaceuticals (Basel). 2023 Dec 21;17(1):14. doi: 10.3390/ph17010014 (PMC10820691; doi:10.3390/ph17010014)
Supplement: Supplementary file 1 [file pharmaceuticals-17-00014-s001.zip › Table S4 .docx]

Table S4: Leave-one-out summary

1. Melanoma

| Study name | IR per 100 PY | Lower CI | Upper CI |
| --- | --- | --- | --- |
| Blauvelt2020 | 0.082 | 0.046 | 0.144 |
| Burmester2020 | 0.089 | 0.052 | 0.154 |
| Burmester2022a | 0.078 | 0.043 | 0.141 |
| Burmester2022b | 0.085 | 0.048 | 0.150 |
| Coates2021 | 0.086 | 0.050 | 0.148 |
| Coates2022 | 0.081 | 0.046 | 0.143 |
| Combe2020 | 0.089 | 0.050 | 0.156 |
| Kivitz2019 | 0.080 | 0.046 | 0.142 |
| Lebwohl2019 | 0.089 | 0.052 | 0.153 |
| Leonardi2020 | 0.089 | 0.051 | 0.152 |
| McInnes2017 | 0.089 | 0.052 | 0.153 |
| Mease2020 | 0.086 | 0.050 | 0.147 |
| Odnopozova2022 | 0.085 | 0.049 | 0.146 |
| Ostor2023 | 0.081 | 0.046 | 0.142 |
| Thaci2021a | 0.087 | 0.048 | 0.157 |
| Thaci2021b | 0.079 | 0.043 | 0.147 |
| Abbreviations: IR, incidence rate; PY, patient-years; CI, confidence interval | | | |

1. Non-melanoma skin cancer

| Study name | IR per 100 PY | Lower CI | Upper CI |
| --- | --- | --- | --- |
| Blauvelt2020 | 0.437 | 0.315 | 0.607 |
| Blauvelt2023 | 0.467 | 0.337 | 0.649 |
| Burmester2020 | 0.445 | 0.321 | 0.617 |
| Burmester2022a | 0.432 | 0.312 | 0.598 |
| Burmester2022b | 0.433 | 0.321 | 0.586 |
| Coates2021 | 0.463 | 0.345 | 0.620 |
| Coates2022 | 0.475 | 0.360 | 0.627 |
| Combe2020 | 0.446 | 0.319 | 0.624 |
| Gossec2023 | 0.480 | 0.364 | 0.632 |
| Kivitz2019 | 0.460 | 0.340 | 0.622 |
| Kristensen2023 | 0.438 | 0.314 | 0.610 |
| Lebwohl2019 | 0.481 | 0.368 | 0.629 |
| Leonardi2020 | 0.465 | 0.346 | 0.626 |
| McInnes2017 | 0.432 | 0.317 | 0.589 |
| Mease2020 | 0.460 | 0.342 | 0.618 |
| Odnopozova2022 | 0.454 | 0.336 | 0.613 |
| Ostor2023 | 0.439 | 0.342 | 0.563 |
| Papp2016 | 0.430 | 0.309 | 0.599 |
| Papp2021 | 0.441 | 0.313 | 0.620 |
| Thaci2021a | 0.444 | 0.316 | 0.624 |
| Thaci2021b | 0.450 | 0.321 | 0.629 |
| Abbreviations: IR, incidence rate; PY, patient-years; CI, confidence interval | | | |
